# Administration of molecular hydrogen during pregnancy improves behavioral abnormalities of offspring in a maternal immune activation model

**DOI:** 10.1038/s41598-018-27626-4

**Published:** 2018-06-15

**Authors:** Kenji Imai, Tomomi Kotani, Hiroyuki Tsuda, Tomoko Nakano, Takafumi Ushida, Akira Iwase, Taku Nagai, Shinya Toyokuni, Akio Suzumura, Fumitaka Kikkawa

**Affiliations:** 1 Department of Obstetrics and Gynecology, Nagoya University Graduate School of Medicine, 65 Tsurumai-cho, Showa-ku, Nagoya, 466-8550 Japan; 2 Department of Neuropsychopharmacology and Hospital Pharmacy, Nagoya University Graduate School of Medicine, 65 Tsurumai-cho, Showa-ku, Nagoya, 466-8550 Japan; 3 Department of Pathology and Biological Responses, Nagoya University Graduate School of Medicine, 65 Tsurumai-cho, Showa-ku, Nagoya, 466-8550 Japan; 4 Department of Neuroimmunology, Research Institute of Environmental Medicine, Nagoya University, Furo-cho, Chikusa-ku, Nagoya, 464-8601 Japan; 5 Department of Obstetrics and Gynecology, Japanese Red Cross Nagoya Daiichi Hospital, 3-35, Michishita-Cho, Nakamura-Ku, Nagoya, 453-8511 Japan

## Abstract

The aim of the present study was to investigate long-term outcomes of the offspring in a lipopolysaccharide (LPS)-induced maternal immune activation (MIA) model and the effect of maternal molecular hydrogen (H_2_) administration. We have previously demonstrated in the MIA mouse model that maternal administration of H_2_ attenuates oxidative damage and neuroinflammation, including induced pro-inflammatory cytokines and microglial activation, in the fetal brain. Short-term memory, sociability and social novelty, and sensorimotor gating were evaluated using the Y-maze, three-chamber, and prepulse inhibition (PPI) tests, respectively, at postnatal 3 or 4 weeks. The number of neurons and oligodendrocytes was also analyzed at postnatal 5 weeks by immunohistochemical analysis. Offspring of the LPS-exposed dams showed deficits in short-term memory and social interaction, following neuronal and oligodendrocytic loss in the amygdala and cortex. Maternal H_2_ administration markedly attenuated these LPS-induced abnormalities. Moreover, we evaluated the effect of H_2_ on LPS-induced astrocytic activation, both *in vivo* and *in vitro*. The number of activated astrocytes with hypertrophic morphology was increased in LPS-exposed offspring, but decreased in the offspring of H_2_-administered dams. In primary cultured astrocytes, LPS-induced pro-inflammatory cytokines were attenuated by H_2_ administration. Overall, these findings indicate that maternal H_2_ administration exerts neuroprotective effects and ameliorates MIA-induced neurodevelopmental deficits of offspring later in life.

## Introduction

The association between maternal immune activation (MIA) and subsequent neurodevelopmental disorders in offspring has become increasingly recognized, with both epidemiological evidence and research findings in various animal models^1–4^. MIA against viral or bacterial infections influences the developing fetal central nervous system (CNS), and increases the risk of schizophrenia and autism spectrum disorder (ASD) later in life^5–7^. It has been suggested that MIA has a strong impact on microglial development, and microglial disturbance disrupts neurogenesis, neuronal migration, and myelination, thus leading to consequent impairments in the brain function of the offspring^2^. Based on these findings, MIA is believed to be a disease primer^1^. It has also been reported that MIA and MIA-induced fetal neuroinflammation stimulate the generation of reactive oxygen species (ROS) and disturbances in pro-inflammatory cytokine production in the fetal brain^8,9^. These changes are reported to lead to direct and indirect death or dysfunction of neuronal and oligodendrocyte cells^10^, which are considered to be the main targets of fetal brain injury^11^.

We have previously suggested that the maternal administration of molecular hydrogen (H_2_) plays a neuroprotective role in the fetal brain against injury caused by oxidative stress and inflammation^12–14^. Ohsawa *et al*. discovered that H_2_ acts as an antioxidant that selectively neutralizes hydroxyl radicals (•OH) and protects the brain from injury^15^. H_2_ reacts with strong oxidants, such as •OH, but remains mild enough to neither disturb the metabolic redox reactions nor affect ROS signaling. Several subsequent studies have explored the therapeutic and preventive effects of H_2_, and have indicated that H_2_ has other properties as well, including anti-inflammatory and anti-apoptotic effects^16,17^. The safety and some promising benefits of H_2_ have already been demonstrated, not only in animal models but also in patients, including those with Parkinson’s disease^18^, diabetes mellitus^19^ and mild cognitive impairment^20^, although no large randomized controlled trials have been conducted to date. Recently, we demonstrated that the administration of H_2_ to pregnant mouse dams significantly increased H_2_ concentration in the fetal brain, through the maternal-fetal interface, in a mouse model of lipopolysaccharide (LPS)-induced MIA^12^. Fetal damage in this MIA model and the effect of maternal H_2_ administration on this damage can be summarized as follows^12^: (1) high mortality rate of MIA fetuses, (2) brain injury associated with elevated levels of pro-inflammatory cytokines, aberrant microglial activation, and oxidative damage in the MIA fetal brains, and (3) attenuation of these adverse outcomes by maternal H_2_ administration. Thus, the protective effect of maternal H_2_ administration on short-term outcomes in the MIA offspring has been demonstrated. However, long-term outcomes of the surviving offspring are yet to be investigated.

Therefore, the present study investigated long-term neurological outcomes in the MIA mouse model and the efficacy of H_2_ administration in pregnant dams, using both histological and behavioral examinations. Astrocytes, which are the most abundant cell population in the CNS, play a key role in normal brain development^21,22^. However, when astrocytes are exposed to maternally-derived cytokines in MIA models, they secrete various cytokines that lead to neurodevelopmental impairments^23^. Moreover, activation of astrocytes is known to be a factor in the prolongation of brain damage, and contributes to the formation of glial scars that limit neuronal plasticity^24^. Thus, in the present study, the MIA-related astrocytic activation and its modification by H_2_ administration were also investigated, both *in vivo* and *in vitro*.

## Results

### Maternal H_2_ administration restored offspring growth

**Figure 1:**
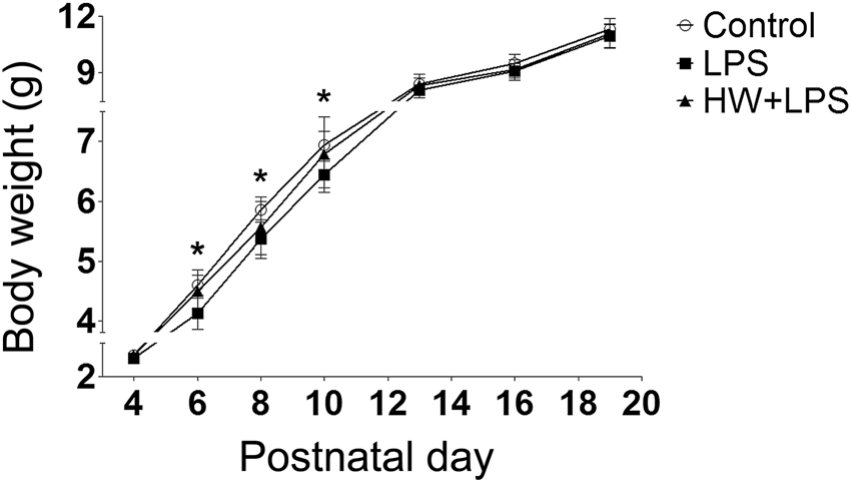
Offspring growth as evidenced by body weight (n = 17–22; Control, 17 pups/5 dams; LPS, 22 pups/5 dams; HW + LPS, 19 pups/5 dams); *p < 0.05, LPS group vs. Control group, by two-way repeated measures ANOVA with Bonferroni post-hoc test. LPS, lipopolysaccharide; HW, hydrogen water.

We investigated postnatal growth, which could be related to neurologic outcome. From postnatal day (P) 6 to P10, offspring growth was significantly retarded in the LPS group than in the Control group (*p* < 0.05). Although significant differences were not observed between the LPS and LPS + HW group, maternal administration of Hydrogen water (HW) restored offspring growth to the control level (Fig. 1).

### Maternal H_2_ administration attenuated the behavioral deficits induced by LPS exposure

**Figure 2:**
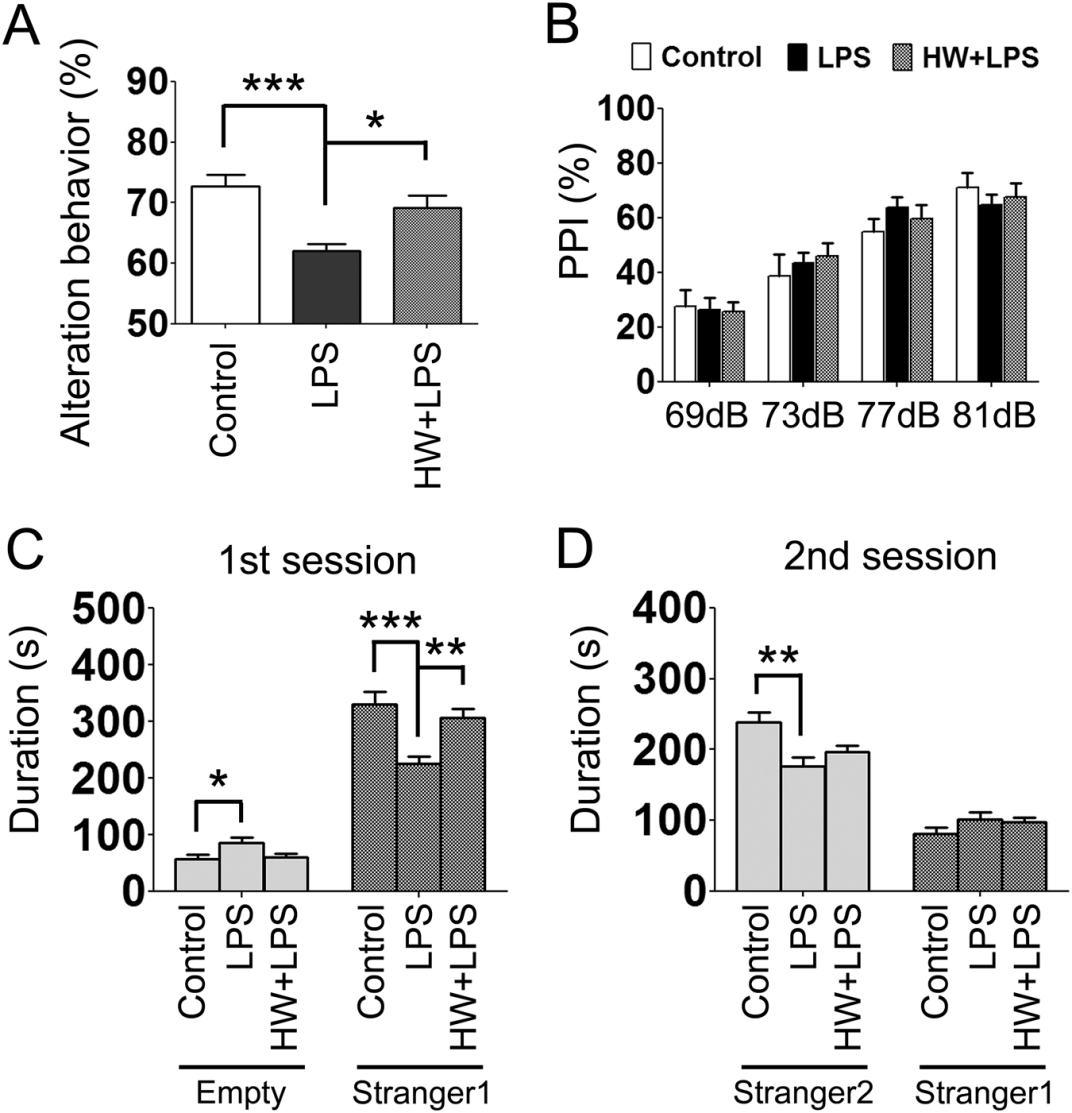
Effects of LPS and H_2_ on the acquisition of short-term recognition memory in the Y-maze test (**A**) PPI test (**B**) and sociability and social novelty preference (**C**,**D**). (**A**) In the LPS group, short-term recognition memory was significantly impaired. In the HW + LPS group, maternal administration of hydrogen water almost completely restored short-term recognition memory. (**B**) No significant difference was detected in each trial. (**C**,**D**) Means ± standard error of the mean are shown for the cumulative data over each 10-min test session. (**C**) Time spent within the sniffing zone of the wire cage containing the novel mouse; Stranger 1, or the empty wire cage. (**D**) Time spent within the sniffing zone of the wire cage containing the familiar mouse; Stranger 1, or Stranger 2 (n = 17–22; Control, 17 pups/5 dams; LPS, 22 pups/5 dams; HW + LPS, 19 pups/5 dams). *p < 0.05, **p < 0.01, ***p < 0.001; by one-way ANOVA with Tukey’s honest significant difference test. LPS, lipopolysaccharide; HW, hydrogen water.

Since MIA has been considered to be a cause of behavioral abnormalities in offspring, including ASD/Schizophrenia-like behavior, we subsequently evaluated the effect of LPS and maternal administration of HW on short-term memory, sociability, social novelty, and sensorimotor gating. As shown in Fig. 2A, spontaneous alternation was markedly reduced in the LPS group than in the Control group (*p* < 0.001) in the Y-maze test, which is indicative of impaired short-term memory. Treatment with H_2_ significantly attenuated the LPS-induced impairment of short-term memory (*p* < 0.05). There was no difference in the total number of arm entries among the three groups (Control, 24.0 ± 1.3; LPS, 22.6 ± 0.61; HW + LPS, 22.7 ± 0.91).

In order to investigate social behaviors, we evaluated sociability and preference for social novelty in the three-chambered social test. During the habituation phase, the mice in each group spent equal amounts of time exploring both compartments, with no biased preference for either of the two empty cylinders (data not shown). During the sociability phase, the mice in each group demonstrated a preference for spending more time in the chamber containing an unfamiliar mouse (stranger 1) relative to the opposite, empty chamber. However, the LPS-treated offspring approached the chamber containing stranger 1 significantly less frequently than the Control and HW + LPS groups (*p* < 0.001 and *p* < 0.01, respectively; Fig. 2C). During the social novelty preference phase, the mice in each group showed a significant preference for the new, unfamiliar mouse (stranger 2) over the previous, now familiar, mouse (stranger 1). However, the LPS-treated offspring approached the chamber containing the stranger 2 significantly less frequently than the Control group (*p* < 0.01; Fig. 2D). There was no difference between the LPS and the HW + LPS groups, although a trend toward attenuation of the LPS-induced reduction in the preference for social novelty was detected in the HW + LPS group.

The prepulse inhibition (PPI) test was performed to evaluate sensorimotor integration by measuring the startle response to administered acoustic pulses, however, no significant differences were detected among the groups (Fig. 2B).

### Maternal H_2_ administration attenuated the LPS-induced neuronal loss

**Figure 3:**
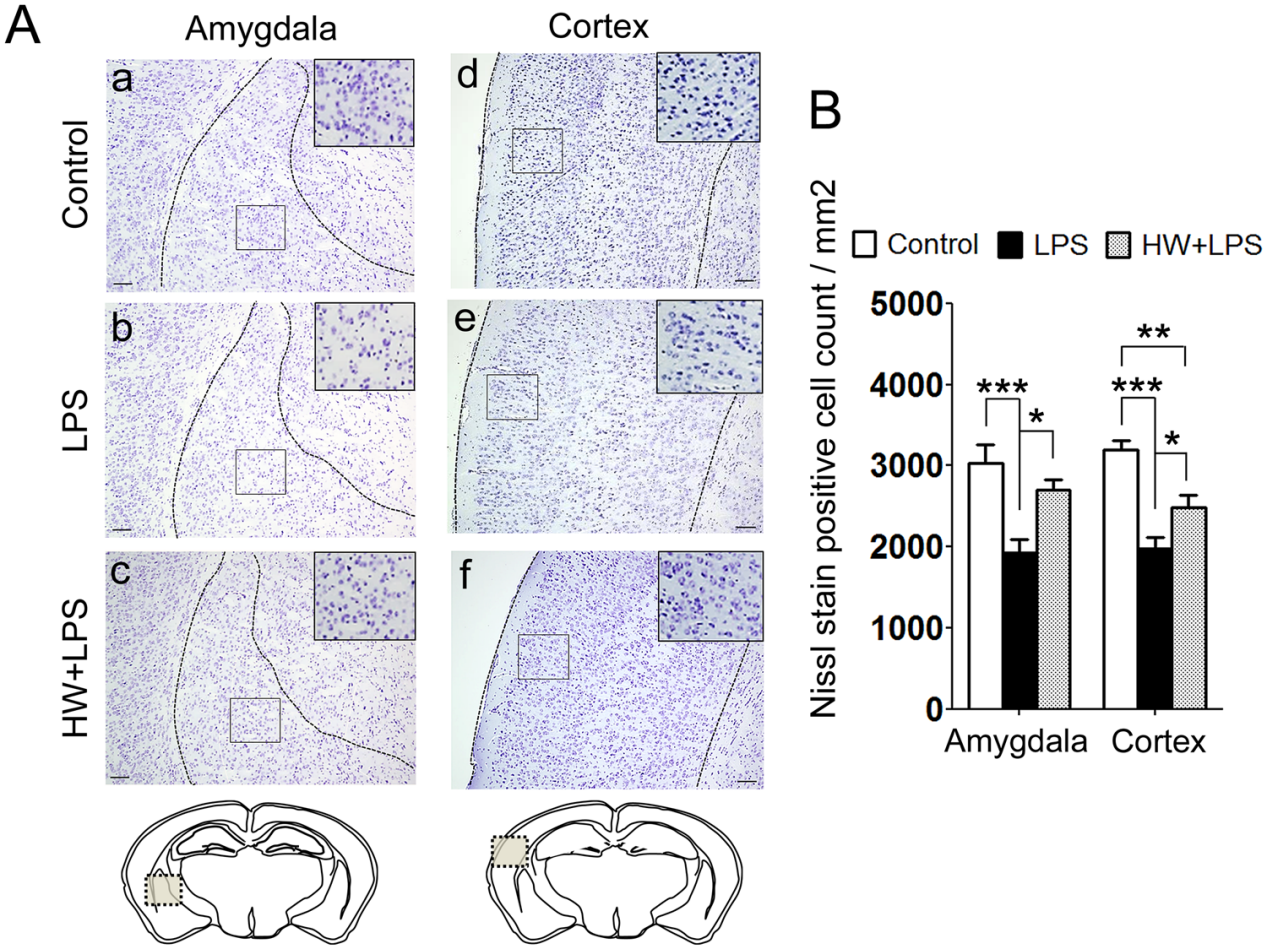
Effect of H_2_ on LPS-induced neuronal loss. (**A**) Representative images of Nissl staining; amygdala and cortex. Scale bar = 100 μm. (**B**) The quantitation of the number of Nissl-positive cells was performed as described in the Materials and Methods. The result is expressed as the mean ± standard error of the mean (n = 12 in each group). Data are analyzed by one-way ANOVA with Tukey’s honest significant difference test. *p < 0.05, **p < 0.01, ***p < 0.001. LPS, lipopolysaccharide.

In our previous investigation, activated microglia was most accumulated in mainly temporal association area, 8 h after the LPS insult. Neurons in this area were reported to be related to sociability deficits^25^. In addition, the association between autism and amygdala, especially in Lateral amygdala^26,27^, is well known. Moreover, the amygdala interacts with the hippocampus in relation to memory^28^. Based on those findings, we subsequently performed Nissl staining and evaluated the number of neurons in the amygdala, cerebral cortex, and hippocampus to investigate the brain regions related to the results of the behavioral deficits induced by LPS exposure. The quantitative results revealed significant reductions in the number of neurons in the amygdala and cerebral cortex in the LPS-treated offspring than in the Control group (*p* < 0.001 and *p* < 0.001, respectively; Fig. 3A, panels a,b,d,e and 3B), but not in the hippocampus (Fig. S1). The LPS-induced neuronal loss in the amygdala and cerebral cortex was improved in the HW + LPS group than in the LPS group (*p* < 0.05 and *p* < 0.05, respectively; Fig. 3A, panels c,f and 3B).

### Maternal H_2_ administration attenuated LPS-induced oligodendrocytic loss and suppressed astrocytic activation

**Figure 4:**
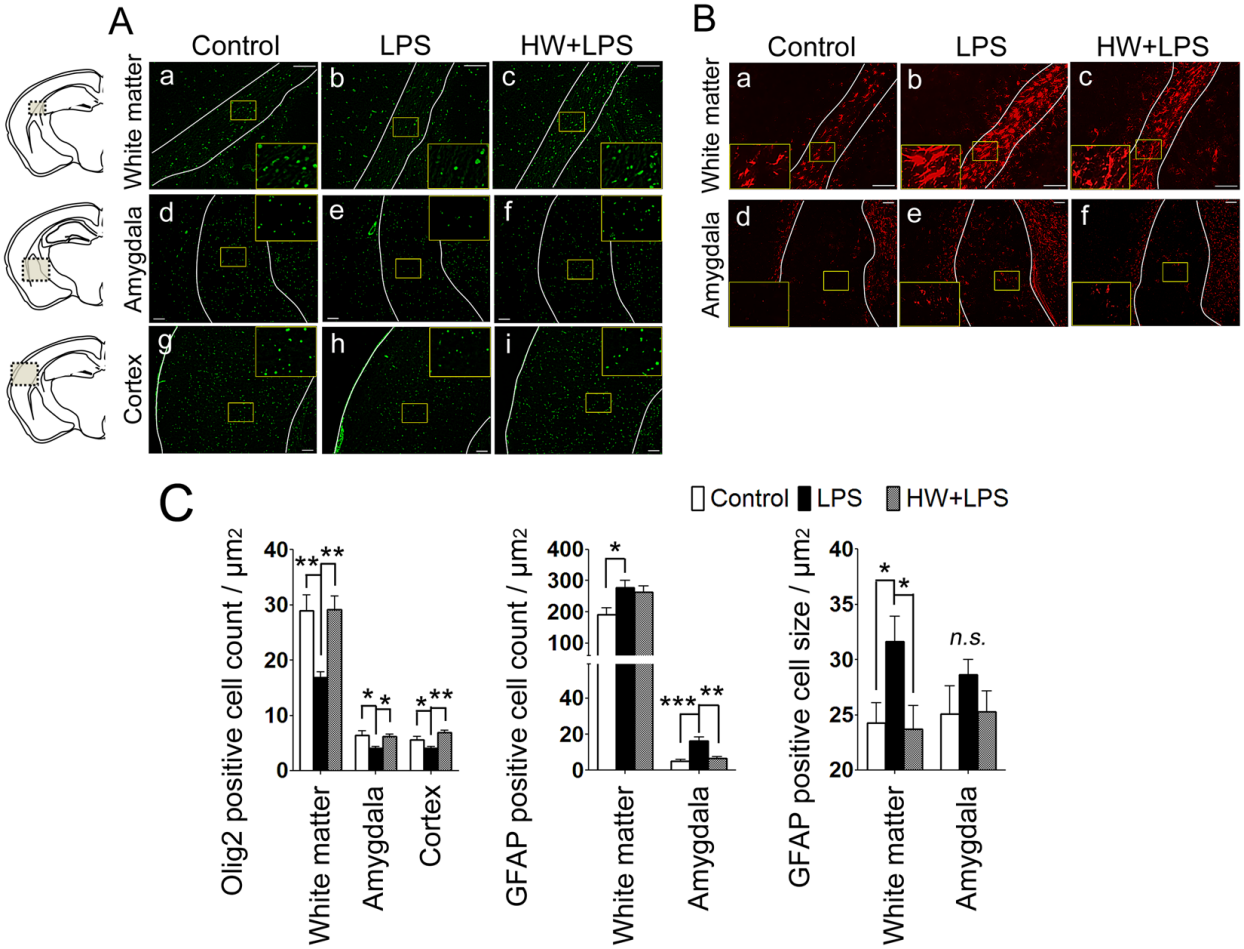
Effect of H_2_ on LPS-induced oligodendrocytic loss and astrocytic activation. (**A**) Representative images of oligodendroglia stained for Olig2; white matter (upper panels), amygdala (center panels), and cortex (lower panels). (**B**) Representative images of astrocytes stained for GFAP; white matter (upper panels), and amygdala (lower panels). Scale bar = 100 μm in each image. (**C**–**E**) The number of oligodendroglia and astrocytes, and the area of the astrocytic cell body (cell size) were quantified as described in the Materials and Methods. Results are expressed as the mean ± standard error of the mean (n = 12 in each group). *p < 0.05, **p < 0.01, ***p < 0.001; by one-way ANOVA with Tukey’s test. GFAP, glial fibrillary acidic protein; LPS, lipopolysaccharide; n.s., not significant. In each representative image, the white line indicates the border of the observation area; white matter, cortex, or amygdala.

The pathophysiologic mechanisms of the brain injury due to MIA are incompletely understood; however, under prenatal inflammation, activated astrocyte was reported to have a potential cause an adverse effect on neurons and oligodendrocyte, which would result in the abnormal behaviors in later life^29^. The number of oligodendrocytes was evaluated by using Olig2, a transcription factor involved in the differentiation of cells in the oligodendroglial lineage, and a mature oligodendrocyte marker^30,31^. Compared with the Control group, the number of Olig2-positive cells was clearly reduced in the white matter, amygdala, and cerebral cortex in the LPS group (*p* < 0.01, *p* < 0.05, and *p* < 0.05, respectively; Fig. 4A, panels a,b,d,e,g,h and 4C). H_2_ treatment completely blocked the LPS-induced decrease of Olig2-positive cells in all three regions (*p* < 0.01, *p* < 0.05, and *p* < 0.01, respectively; Fig. 4A, panels c,f,i and 4C).

The hallmarks of astrocytic activation include the development of a hypertrophic morphology with fewer processes and upregulation of intermediate filament proteins, particularly glial fibrillary acidic protein (GFAP)^32,33^. In the Control group, some GFAP-positive cells were detected, most of which were in the resting state (Fig. 4B, panels a,d). Compared with the Control group, a significantly increased number of activated astrocytes, characterized by a large soma and fewer processes (thus obtaining a more rounded shape), was observed in the white matter and amygdala of the LPS-treated offspring (*p* < 0.05 and *p* < 0.001, respectively; Fig. 4B, panel b,e and 4D). To evaluate the hypertrophic soma, the cellular size of the astrocytes was also quantified by calculating the ratio of GFAP immunostained area to the number of GFAP-positive cells. Larger soma were observed in the white matter of LPS-exposed mouse brains (*p* < 0.05; Fig. 4B, panel b and 4E). Although the number of GFAP-positive cells was unchanged following H_2_ treatment, the size of the GFAP-positive cells was significantly reduced in the white matter (*p* < 0.05; Fig. 4B, panel c and 4E), indicating a reduced number of reactive astrocytes by H_2_ treatment. In the amygdala, the number of GFAP positive astrocytes in the HW + LPS group was significantly decreased (*p* < 0.01; Fig. 4B, panel f and 4D).

### H_2_ treatment suppressed LPS-induced activation in primary cultured astrocyte

**Figure 5:**
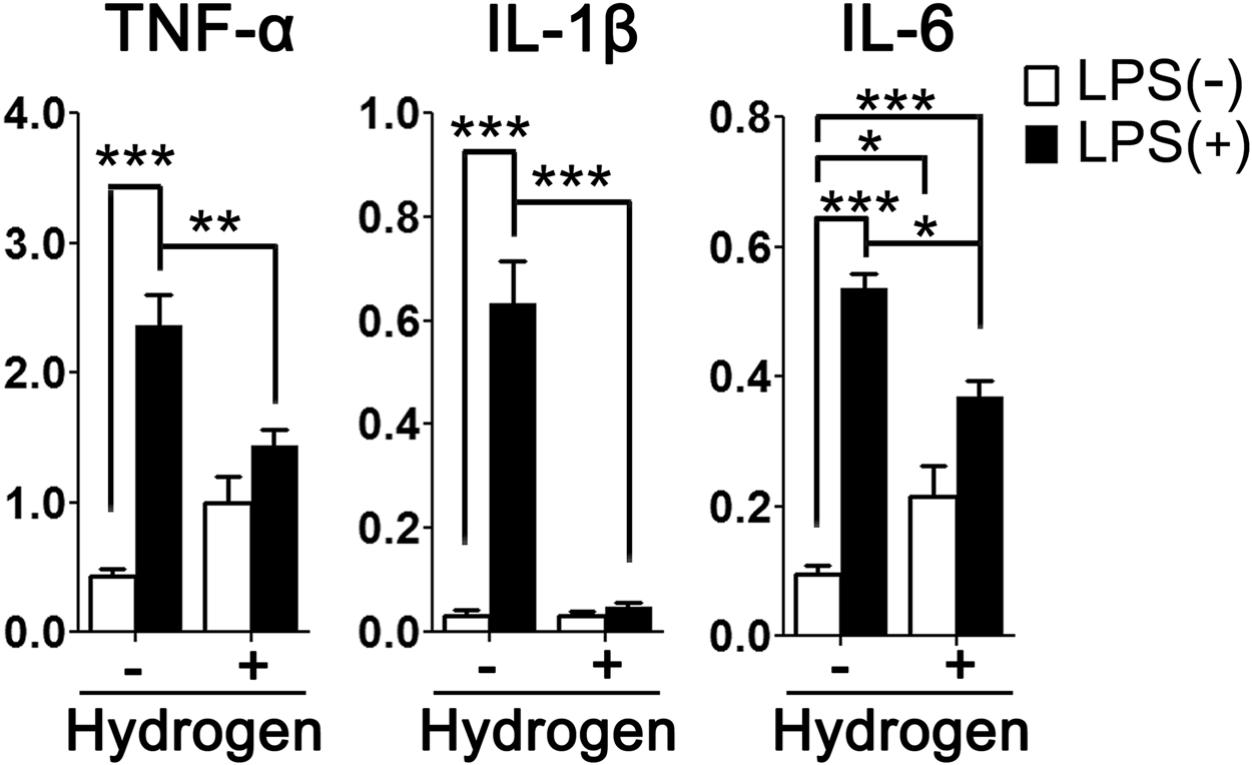
Effect of H_2_ on LPS-induced expression of pro-inflammatory cytokines in primary cultured astrocytes. mRNA expression levels of TNF-α, IL-1β, and IL-6 were determined by qRT-PCR 3 h after the addition of LPS. The data were normalized to the expression of β-actin. Results are expressed as the mean ± standard error of the mean (n = 5 in each group). The data are analyzed by two-way ANOVA, followed by Bonferroni *post*-*hoc* test. *p < 0.05, **p < 0.01, ***p < 0.001. IL, interleukin; LPS, lipopolysaccharide; qRT-PCR, quantitative reverse transcription polymerase chain reaction; TNF, tumor necrosis factor.

Because the over-activation of immune cells, including astrocytes, could have an adverse effect on neurons with the overproduction of pro-inflammatory cytokines^34^, we investigated the anti-inflammatory effect of H_2_ on astrocyte using primary cultured astrocyte. As shown in Fig. 5, stimulation of astrocytes with LPS for three hours led to marked increases in tumor necrosis factor α (TNF-α) (*p* < 0.001), interleukin (IL)-1β (*p* < 0.001), and IL-6 (*p* < 0.001) mRNA expression. These increases in expression of TNF-α (*p* < 0.01), IL-1β (*p* < 0.001) and IL-6 (*p* < 0.05) were significantly attenuated by H_2_ treatment.

### Altered gene expression patterns in primary cultured astrocytes from the Control group, the LPS group, and the HW + LPS group

As we previously described the gene expression profile of microglia^12^, differential gene expression patterns of astrocytes from newborn mice in the Control group, the LPS group, and the HW + LPS group were also observed (Tables S1–S4, Supplementary Information). These results suggest that H_2_ might also influence the function not only of microglia but also of astrocytes via changes in gene expression.

## Discussion

For the first time, we have demonstrated that maternal H_2_ administration improved MIA-induced neurological impairments of offspring, including short-term memory and social interaction at postnatal 3–5 weeks. Previously, we reported that maternal H_2_ administration suppresses microglial activation in the same model. In the present study, H_2_ also suppressed the activation of astrocytes, both *in vitro* and *in vivo*. Taken together, these results suggest that H_2_ exerts a protective role against MIA-induced neuroinflammation through the suppression of microglia and astrocytes. As a result, mature oligodendrocytes were restored and neurons were protected from damage, thus leading to improved long-term neurological outcomes following maternal H_2_ administration. Prenatal exposure to LPS also resulted in a transient reduction in body weight of the offspring between P6 - P10, which was attenuated by maternal H_2_ administration. This might be due to a change in the activity of the hypothalamic-pituitary axis^35^. Deficient post-neonatal growth has been reported to contribute to poor neurologic outcome^36^, thus, the transient decrease of weight observed in this model might be related to the neurological impairments in the offspring.

On the basis of the epidemiological evidence, several translational rodent models have been established to investigate a potential causal relationship between MIA and ASD-like behavioral abnormalities^1^. Following the discovery, in the 1980s, of the increased risk of schizophrenia after maternal influenza infection^37^, models of viral-like immune activation by polyriboinosinic-polyribocytidilic acid (poly(I:C)) have been widely studied^33,38,39^. In addition, models of bacterial-like immune activation by LPS are also well known to precipitate inflammatory responses and behavioral abnormalities in offspring, similar to the poly(I:C) models^38^. However, with respect to neurotoxicity, LPS exposure may have a stronger effect on the fetal brain than poly(I:C) exposure^29^. Thus, the type of pathogen is important for the pattern of neurodevelopmental abnormalities of the offspring, and the timing and dose of the pathogen’s administration, in addition to the strain or species, may also have an influence. The present model of prenatal exposure to LPS led to impairments in short-term memory and social interaction at 3 to 4 weeks after birth.

Alterations in short-term memory were demonstrated by the marked reduction in alternation behavior in the Y-maze test, which is consistent with previous reports^40,41^ and is a persistent finding in poly(I:C) models^38^. The Y-maze test is well known as a hippocampal-dependent short-term learning task. In our MIA model, a remarkable loss of neurons was observed in the amygdala and cerebral cortex of the offspring, but not in the hippocampus. The formation of memory is encoded in a broad network of cortical/subcortical regions including the hippocampus, cingulate cortex, and amygdala^42^. Moreover, the amygdala is directly connected to and dynamically interacts with the hippocampus in relation to memory^28^. Therefore, a decreased number of neurons in the amygdala and cerebral cortex could consequently result in short-term memory impairments. In this model, increased activation of astrocytes was observed in the white matter and amygdala, which be related to impaired short-term memory^28,41^.

Previous reports have demonstrated a causal relationship between prenatal exposure to LPS and deficits in social behavior and sensorimotor gating in offspring^38,43–46^. In this model, the social behavior including sociability and social novelty preference was impaired. The number of oligodendrocytes in the amygdala, cerebral cortex, and white matter in the offspring was decreased, which is consistent with previous findings^47^; and a reduced number of neurons in the amygdala and cortex was also observed. Strong evidence suggests a crucial involvement of the amygdala in social processing and social cognition in humans^48–50^, as well as in social behavior in animals^51–53^. A recent report also suggested an association between the function of the cortex and the amygdala, and social behavior^54^. Therefore, the social behavior impairment observed in the present study is compatible with the neuronal and oligodendrocytic loss in the amygdala and cortex. A locomotor test was not performed in this study, although we observed no apparent differences in speed of movement among all groups in the three-chamber social test. Interestingly, there were no remarkable differences in the PPI test in our MIA model, and sensorimotor gating was also normal in comparison with the Control group. A possible explanation for these results is that PPI abnormalities might occur following exposure to a pathogen at mid-gestation^55^, while impairments in short-term memory and social behavior might be independent of the timing of exposure to a pathogen^2^. Thus, the negative results of the PPI test might be due to the fact that in the present model, LPS exposure occurs at late gestation, on embryonic day 17 (E17). Impairments in short-term memory and social behavior are well described in schizophrenia and ASD^38,56^. PPI impairments manifest primarily in adults with ASD^57^, and are not observed in children with ASD^58^. A recent study further suggested that sensorimotor gating is only impaired in certain ASD subgroups^59^, although it may be a globally common feature in schizophrenia^60^. Therefore, the present model may reflect ASD-like rather than schizophrenia-like behavioral impairments.

Several characteristics that are reported in human ASD brains were also observed in the present model, including neuronal loss in the amygdala and cerebral cortex^61^, and astrocytic activation in the white matter^62^. We and others have also previously demonstrated microglial activation, enhanced pro-inflammatory cytokine levels, including IL-6, and elevated oxidative damage in fetal brains^12,63–65^. In the MIA model, microglia play a major role, along with astrocytes, in synaptic pruning and neural circuit formation^2^. In addition, a single injection of IL-6 is known to lead to the same behavioral abnormalities as those observed in the LPS models^66^ (IL-6 is thought to be a key cytokine in the link between MIA and the behavioral deficits in the offspring^38^). In our previous report, maternal H_2_ administration reduced both the excessive microglial activation and the increased IL-6 levels in the MIA fetal brains^12^, which could ameliorate the behavioral deficits later in life. Previous studies in adult rodents reported that H_2_ treatment could mitigate the behavioral dysfunctions, including short-term memory deficits, cognitive impairments, and depressive-like behaviors, by regulating inflammation and ROS production^67,68^, which is in line with our current results. Moreover, the present study demonstrated that prenatal H_2_ administration restored the MIA-induced neuronal and oligodendrocytic loss, and suppressed the excessive activation of astrocytes at postnatal 5 weeks. It was also shown that H_2_ directly attenuated the LPS-induced expression of pro-inflammatory cytokines, including TNF-α, IL-1β, and IL-6, in primary cultured astrocytes. These cytokines are known to induce apoptosis in oligodendrocytes and hypomyelination in the neonatal rodent^69^. Thus, the suppressive effect of H_2_ on both microglial and astrocytic activation would protect neurons and oligodendrocytes against LPS exposure.

The pathological mechanism by which MIA causes psychiatric diseases in offspring is completely unknown, however we speculate that the following steps may be involved^29,70^. Intrauterine inflammation activates fetal microglia during the acute phase, and subsequently, microglia also activate astrocytes. In the chronic phase, microglial activation is attenuated, while astrocytes are continuously activated^29^. Both microglia and astrocytes are important for normal neurodevelopment, however under prenatal inflammation, they are activated, their function is altered, and they have an adverse effect on neurons and axons, with the overproduction of pro-inflammatory cytokines^34^. Inflammation also prevents the maturation of oligodendroglial progenitor cells, followed by a reduction of the oligodendrocyte lineage^71,72^. This reduction leads to axonal loss. The neuronal and axonal damage caused by microglia or astrocytes in the developing brain would result in the abnormal behavioral features in later life. In our model, we have previously reported over-activation of fetal microglia 8 h after the LPS insult^12^, and the present study demonstrated that activation of astrocytes continued even ~5 weeks after the insult. Moreover, the number of neurons and oligodendrocytes were reduced in the amygdala and white matter, respectively. We have previously reported that H_2_ has a suppressive effect on microglial activation^12^, and the present study demonstrated that H_2_ had a similar effect on astrocytes, as described above. From these findings, we speculate that H_2_ would protect neurons and oligodendrocytes indirectly via suppression of the over-activation of immune cells, including microglia and astrocytes, which would result in a reduction of the behavioral abnormalities present in MIA offspring. However, in this study, a direct effect of H_2_ on neurons and oligodendrocytes was not evaluated *in vitro*, thus, this possibility cannot be excluded.

Several limitations of this study should be noted. In the current MIA model, additional core features of ASD, including restricted and repetitive behavioral patterns, were not investigated^73^. Thus, the effect of maternal H_2_ administration on these symptoms remains unknown. The sensorimotor gating abnormality may be evaluated at an older age than was described in the present model, however, this might require changing other factors including the timing of exposure. Since the mouse brain is not considered to be fully mature until 3 weeks of age^74^, the interpretation of the results of behavioral tests in the present study requires considerable caution. In addition, we could not avoid the detrimental effects of maternal separation required for the measurement of body weight on offspring development. However, the separation was performed similarly for the offspring in all three groups, thus any effect should be minimal, and consistent across groups. In humans, it is currently thought that the occurrence of multiple risk factors, including genetic mutations and postnatal exposure to inflammation or other environmental triggers, may be required for the onset of ASD^1,38^. Before advancing the clinical application of H_2_, it should be investigated whether H_2_ could exert similar effects in so called ‘multi-hit’ models. Finally, in the previous and present studies, maternal H_2_ administration was performed prior to the LPS exposure; thus, the therapeutic potential of H_2_ remains unclear. Therefore, it might be relevant to evaluate the therapeutic effects of H_2_ by administering H_2_ to dams after exposure to LPS and in offspring.

In conclusion, we have provided evidence that maternal H_2_ administration reduced deficits in short-term memory and social interaction in the LPS-induced MIA model, and also demonstrated an attenuation of the LPS-induced neuronal and oligodendrocytic cell loss. Remarkably, the neuroprotective effect of H_2_ appeared in the amygdala and cortex in this mouse model. The neuroprotection is thought to be due to the suppression of neuroinflammation, including excessive activation of microglia and astrocytes and the increased level of pro-inflammatory cytokines, as reported previously^12^. Although further investigation is required, our current and previous results point to the potential efficacy of maternal H_2_ administration on the long-term neurological outcomes of offspring exposed to inflammation *in utero*.

## Materials and Methods

### Reagents

LPS (serotype O55:B5) was obtained from Sigma-Aldrich (St. Louis, MO, USA). HW and hydrogen medium (HM) were prepared by dissolving H_2_ gas as described previously^12,75^. Both HW and HM had a concentration of >0.4 mM H_2_.

### Animals and treatments

**Figure 6:**
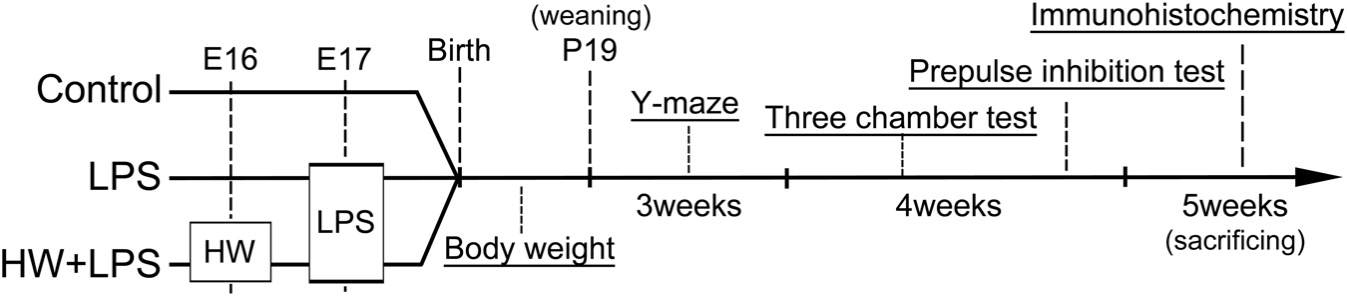
Schematic timeline of the *in vivo* study design and experimental protocols.

The experimental protocols in this study were approved by the Animal Experiment Committee of Nagoya University (approval number: 29154), and were carried out in accordance with the Regulations on Animal Experiments in Nagoya University. Pregnant ICR (CD-1) mice (7–8 weeks) were purchased from Charles River Laboratories (Kanagawa, Japan). All mice were allowed free access to food and water and were maintained on a 12-h light/dark cycle (lights on at 9:00 AM). The pregnant mice were assigned randomly to three groups of five dams each: Control group, LPS group, and HW + LPS group (Fig. 6), as in our previous report^12^. All pregnant mice, except for those in the Control group, received an intraperitoneal injection (i.p.) of 75 μg of LPS dissolved in sterile saline on E17. In the Control group, the pregnant mice were injected with an equal volume of sterile saline. In the HW + LPS group, the pregnant mice were administered HW, beginning 24 h before LPS injection and continuing until parturition. HW was aliquoted in glass drinking bottles to prevent H_2_ degassing as well as air refilling.

### Offspring growth evaluation and behavioral testing

As shown in Fig. 6, after birth, all offspring were housed with their mother until P19, when they were weaned, as previously reported^76,77^. We only used male offspring in all of the experiments because of previously reported sex effects^43^. All animals were left undisturbed except for measuring body weights and weekly cage changes. Body weight was recorded at P4, 6, 8, 10, 13, 16, and 19. The order of the behavioral tests was as follows: (1) Y-maze test at postnatal 3 weeks^78^; and (2) three-chamber sociability and social novelty test^79^ and PPI test at postnatal 4 weeks, according to previous reports (Fig. 6). All behavioral tests were conducted between 1 PM and 8 PM.

#### Y-maze test

The Y-maze test was carried out as described previously^80^. Briefly, the test was performed in a Y-shaped maze with three opaque plastic arms situated at 120° angles from each other. Each mouse was placed individually in the center of the apparatus and allowed to explore the maze freely during an 8-min session. The series of arm entries were recorded visually. Alternation was defined as successive entries into the three arms, on overlapping triplet sets. The percent alternation was calculated as the ratio of actual to possible alternations (defined as the total number of arm entries minus two) multiplied by 100. Spontaneous alternation (%) was used to quantify short-term memory.

#### Three-chamber social test

The three-chamber social test was performed as described previously^81^. Briefly, the apparatus consisted of a black Plexiglas rectangular box and two identical clear Plexiglas cylinders. There were three interconnected chambers in the box. The light was conditioned at 20 lux in an experimental room. During the habituation phase, empty cylinders were placed in each of the end chambers. A mouse was introduced to the center chamber and its behavioral approach to the end chambers was monitored for 10 min. During the sociability test, an unfamiliar male ICR (CD-1) mouse (stranger 1) that had no prior contact with the test mouse was placed in one of the empty chambers, and the behavioral approach of the test mouse to the empty chamber and stranger 1 was monitored for 10 min. During the social novelty test, a new unfamiliar male ICR (CD-1) mouse (stranger 2) was placed in the third chamber, and the behavioral approach of the test mouse to stranger 1 and stranger 2 was monitored for 10 min. The time spent in each zone was calculated using the Ethovision automated tracking program (Noldus, Wageningen, The Netherlands).

#### Prepulse inhibition test

The PPI test was carried out as described previously^80^ with the SR-LAB system (San Diego Instruments, San Diego, California). Briefly, after habituation for 10 min, the animals received 10 startle trials, 10 no-stimulus trials, and 40 PPI trials. Each startle trial consisted of a single 120 dB white noise burst lasting 40 msec. The PPI trials consisted of a prepulse (20 msec burst of white noise at 69, 73, 77, or 81 dB intensity) followed, 100 msec later, by the startle stimulus (120 dB, 40 msec white noise). The total session lasted 17 min. The resulting movement of the animal in the startle chamber was measured for 100 msec after the startle stimulus onset (sampling frequency 1 kHz), rectified, amplified, and input into a computer, which calculated the maximal response over the 100-msec period. The basal startle amplitude was determined as the mean amplitude of the 10 startle trials. The PPI was calculated according to the following formula: 100 × [1 − (PP×/P120)] %, in which PPx was the mean of the 10 PPI trials (PP69, PP73, PP75, or PP81) and P120 was the basal startle amplitude.

### Histological analysis

After the behavioral analysis, 12 offspring in each group were selected randomly and sacrificed by decapitation at postnatal 5 weeks (Fig. 6). The offspring were perfused via the heart with 4% paraformaldehyde (PFA) in PBS. The brains were then fixed in 4% PFA for at least 24 h, embedded in paraffin, and cut in coronal planes (approximately 2.0 mm posterior to bregma). Immunofluorescence staining was performed using the following primary antibodies: rabbit monoclonal anti-Olig2 antibody (ab109186, 1:100; Abcam, Tokyo, Japan) or rabbit polyclonal anti-GFAP antibody (23935-1-AP, 1:100; Proteintech, Chicago, IL). The sections were incubated with the primary antibodies at 4 °C overnight and were further incubated with secondary antibodies (Alexa fluor 488, 1:500 or Alexa fluor 563, 1:500; Invitrogen, Carlsbad, CA) for 30 min at room temperature. Nissl staining was also performed in order to evaluate the number of neuronal cells. Following deparaffinization, the tissue sections were incubated in 0.2% cresyl violet (Muto Pure Chemicals Co.), and subsequently dehydrated in graded ethanol, and permanently mounted. Images were acquired with a BZ-9000 microscope (Keyence Corporation, Osaka, Japan). The BZ Image Measurement Software program (Keyence) was used to quantify the number of neuronal cells and Olig2-positive cells, as well as the number and the soma size of the GFAP-positive cells.

### Astrocytic cultures

Mouse primary astrocytes were isolated from primary mixed glial-cell cultures of newborn ICR mice, as described previously^82,83^. The purity of the astrocytes was >95%, as determined by immunostaining with the anti-GFAP antibody. Astrocytes were plated at a density of 3 × 10^5^ cells/well in 6-well dishes. Cells were maintained in Dulbecco’s modified Eagle’s minimum essential medium (Sigma-Aldrich) supplemented with 10% fetal bovine serum, 5 mg/ml bovine insulin, and 0.2% glucose. To assess the expression of pro-inflammatory cytokines, confluent astrocytes were treated with 1 μg/ml LPS and/or HM. H_2_ treatment was performed by replacing the astroglial medium with HM, and dehydrogenated HM was used as a control medium. The time course of H_2_ concentration in HM was described previously^12^. Replacement of the astroglial medium with HM or control medium was performed 3 h before the addition of LPS.

### Quantitative reverse transcription-polymerase chain reaction (RT-PCR)

Total RNA from the astrocyte cultures was extracted using the RNeasy Mini Kit (Qiagen Inc., Tokyo, Japan). The RT reaction with 100 ng of total RNA was carried out with a first strand cDNA synthesis kit (ReverTra Ace; Toyobo Co., Ltd, Osaka, Japan). The expression levels of mRNAs encoding TNF-α, IL-1β, and IL-6 were evaluated by quantitative PCR (qPCR) using the Thermal Cycler Dice (Takara Bio Inc., Tokyo, Japan) and SYBRII Premix Ex Taq (Takara Bio Inc.) reagents. Primers for qRT-PCR are listed in Table 1.

**Table 1:** List of primers for qRT-PCR.

Genes	Primer sequences	
	Forward	Reverse
TNF-α	5′-GTAGCCCACGTCGTAGCAAAC-3′	5′-CTGGCACCACTAGTTGGTTGTC-3′
IL-6	5′-ACAACCACGGCCTTCCCTAC-3′	5′-TCCACGATTTCCCAGAGAACA-3′
IL-1β	5′-CATCCAGCTTCAAATCTCGCAG-3′	5′-CACACACCAGCAGGTTATCATC-3′
β-actin	5′-CGTGGGCCGCCCTAGGCACCA-3′	5′-ACACGCAGCTCATTGTA-3′

### Statistical analysis

The data are presented as means ± standard error of the mean (SEM). To analyze the neonatal body weights, two-way repeated measures analysis of variance (ANOVA) was used, followed by a Bonferroni *post*-*hoc* test. The mRNA expression levels in astrocyte cultures were also analyzed by two-way ANOVA, followed by a Bonferroni *post*-*hoc* test. All other data were analyzed using one-way ANOVA followed by Tukey’s test. The statistical analyses were performed using Prism 5 for Windows (GraphPad Software, San Diego, CA). Values of *p* < 0.05 were considered to be statistically significant.

## Electronic supplementary material

Supplementary information

## References

[1] Estes ML, McAllister AK (2016). Maternal immune activation: Implications for neuropsychiatric disorders. Science (New York, N.Y.).

[2] Knuesel I (2014). Maternal immune activation and abnormal brain development across CNS disorders. Nat Rev Neurol.

[3] Bilbo, S. D., Block, C. L., Bolton, J. L., Hanamsagar, R. & Tran, P. K. Beyond infection - Maternal immune activation by environmental factors, microglial development, and relevance for autism spectrum disorders. *Exp Neurol*, 10.1016/j.expneurol.2017.07.002 (2017). 10.1016/j.expneurol.2017.07.002 PMC5723548 28698032

[4] Scola G, Duong A (2017). Prenatal maternal immune activation and brain development with relevance to psychiatric disorders. Neuroscience.

[5] Khandaker GM, Zimbron J, Lewis G, Jones PB (2013). Prenatal maternal infection, neurodevelopment and adult schizophrenia: a systematic review of population-based studies. Psychol Med.

[6] Patterson PH (2011). Maternal infection and immune involvement in autism. Trends Mol Med.

[7] Brown AS (2012). Epidemiologic studies of exposure to prenatal infection and risk of schizophrenia and autism. Dev Neurobiol.

[8] Volpe JJ (2009). Brain injury in premature infants: a complex amalgam of destructive and developmental disturbances. Lancet Neurology.

[9] Haynes RL (2003). Nitrosative and oxidative injury to premyelinating oligodendrocytes in periventricular leukomalacia. Journal of Neuropathology and Experimental Neurology.

[10] Yuan TM, Sun Y, Zhan CY, Yu HM (2010). Intrauterine infection/inflammation and perinatal brain damage: Role of glial cells and Toll-like receptor signaling. Journal of Neuroimmunology.

[11] Malaeb S, Dammann O (2009). Fetal Inflammatory Response and Brain Injury in the Preterm Newborn. Journal of Child Neurology.

[12] Imai K (2016). Neuroprotective potential of molecular hydrogen against perinatal brain injury via suppression of activated microglia. Free Radic Biol Med.

[13] Nakano T (2015). Maternal molecular hydrogen administration on lipopolysaccharide-induced mouse fetal brain injury. J Clin Biochem Nutr.

[14] Mano Y (2014). Maternal molecular hydrogen administration ameliorates rat fetal hippocampal damage caused by in utero ischemia-reperfusion. Free Radic Biol Med.

[15] Ohsawa I (2007). Hydrogen acts as a therapeutic antioxidant by selectively reducing cytotoxic oxygen radicals. Nat Med.

[16] Ohta S (2014). Molecular hydrogen as a preventive and therapeutic medical gas: initiation, development and potential of hydrogen medicine. Pharmacol Ther.

[17] Ichihara M (2015). Beneficial biological effects and the underlying mechanisms of molecular hydrogen - comprehensive review of 321 original articles. Medical gas research.

[18] Yoritaka A (2013). Pilot study of H(2) therapy in Parkinson’s disease: a randomized double-blind placebo-controlled trial. Mov Disord.

[19] Kajiyama S (2008). Supplementation of hydrogen-rich water improves lipid and glucose metabolism in patients with type 2 diabetes or impaired glucose tolerance. Nutr Res.

[20] Nishimaki, K. *et al*. Effects of molecular hydrogen assessed by an animal model and a randomized clinical study on mild cognitive impairment. *Current Alzheimer research*, 10.2174/1567205014666171106145017 (2017). 10.2174/1567205014666171106145017 PMC5872374 29110615

[21] Parpura V (2012). Glial cells in (patho)physiology. Journal of Neurochemistry.

[22] Araque A (2014). Gliotransmitters Travel in Time and Space. Neuron.

[23] Ibi D, Yamada K (2015). Therapeutic Targets for Neurodevelopmental Disorders Emerging from Animal Models with Perinatal Immune Activation. International Journal of Molecular Sciences.

[24] Wasielewski B, Jensen A, Roth-Harer A, Dermietzel R, Meier C (2012). Neuroglial activation and Cx43 expression are reduced upon transplantation of human umbilical cord blood cells after perinatal hypoxic-ischemic injury. Brain Research.

[25] Shin Yim Y (2017). Reversing behavioural abnormalities in mice exposed to maternal inflammation. Nature.

[26] Schumann CM, Amaral DG (2006). Stereological analysis of amygdala neuron number in autism. J Neurosci.

[27] West MJ, Slomianka L, Gundersen HJ (1991). Unbiased stereological estimation of the total number of neurons in thesubdivisions of the rat hippocampus using the optical fractionator. The Anatomical record.

[28] Richter-Levin G, Akirav I (2000). Amygdala-hippocampus dynamic interaction in relation to memory. Mol Neurobiol.

[29] Hagberg H, Gressens P, Mallard C (2012). Inflammation during fetal and neonatal life: implications for neurologic and neuropsychiatric disease in children and adults. Ann Neurol.

[30] Yokoo H (2004). Anti-human olig2 antibody as a useful immunohistochemical marker of normal oligodendrocytes and gliomas. American Journal of Pathology.

[31] Zhou Q, Choi G, Anderson DJ (2001). The bHLH transcription factor Olig2 promotes oligodendrocyte differentiation in collaboration with Nkx2.2. Neuron.

[32] Fan LW (2013). Celecoxib attenuates systemic lipopolysaccharide-induced brain inflammation and white matter injury in the neonatal rats. Neuroscience.

[33] Coyle P, Tran N, Fung JN, Summers BL, Rofe AM (2009). Maternal dietary zinc supplementation prevents aberrant behaviour in an object recognition task in mice offspring exposed to LPS in early pregnancy. Behav Brain Res.

[34] Ratnayake U, Quinn T, Walker DW, Dickinson H (2013). Cytokines and the neurodevelopmental basis of mental illness. Front Neurosci.

[35] Izvolskaia MS, Tillet Y, Sharova VS, Voronova SN, Zakharova LA (2016). Disruptions in the hypothalamic-pituitary-gonadal axis in rat offspring following prenatal maternal exposure to lipopolysaccharide. Stress (Amsterdam, Netherlands).

[36] Guellec I (2016). Effect of Intra- and Extrauterine Growth on Long-Term Neurologic Outcomes of Very Preterm Infants. The Journal of pediatrics.

[37] Mednick SA, Machon RA, Huttunen MO, Bonett D (1988). Adult schizophrenia following prenatal exposure to an influenza epidemic. Arch Gen Psychiatry.

[38] Meyer U (2014). Prenatal poly(i:C) exposure and other developmental immune activation models in rodent systems. Biol Psychiatry.

[39] Chlodzinska N, Gajerska M, Bartkowska K, Turlejski K, Djavadian RL (2011). Lipopolysaccharide injected to pregnant mice affects behavior of their offspring in adulthood. Acta Neurobiol Exp (Wars).

[40] Chen GH (2011). Acceleration of age-related learning and memory decline in middle-aged CD-1 mice due to maternal exposure to lipopolysaccharide during late pregnancy. Behav Brain Res.

[41] Yin P (2015). Maternal immune activation increases seizure susceptibility in juvenile rat offspring. Epilepsy & behavior: E&B.

[42] Zhang Y, Fukushima H, Kida S (2011). Induction and requirement of gene expression in the anterior cingulate cortex and medial prefrontal cortex for the consolidation of inhibitory avoidance memory. Mol Brain.

[43] Xuan IC, Hampson DR (2014). Gender-dependent effects of maternal immune activation on the behavior of mouse offspring. Plos One.

[44] Wischhof L, Irrsack E, Dietz F, Koch M (2015). Maternal lipopolysaccharide treatment differentially affects 5-HT(2A) and mGlu2/3 receptor function in the adult male and female rat offspring. Neuropharmacology.

[45] Wischhof L, Irrsack E, Osorio C, Koch M (2015). Prenatal LPS-exposure–a neurodevelopmental rat model of schizophrenia–differentially affects cognitive functions, myelination and parvalbumin expression in male and female offspring. Prog Neuropsychopharmacol Biol Psychiatry.

[46] Oskvig DB, Elkahloun AG, Johnson KR, Phillips TM, Herkenham M (2012). Maternal immune activation by LPS selectively alters specific gene expression profiles of interneuron migration and oxidative stress in the fetus without triggering a fetal immune response. Brain, behavior, and immunity.

[47] Wang X, Rousset CI, Hagberg H, Mallard C (2006). Lipopolysaccharide-induced inflammation and perinatal brain injury. Semin Fetal Neonatal Med.

[48] Bickart KC, Wright CI, Dautoff RJ, Dickerson BC, Barrett LF (2011). Amygdala volume and social network size in humans. Nat Neurosci.

[49] Felix-Ortiz AC, Tye KM (2014). Amygdala inputs to the ventral hippocampus bidirectionally modulate social behavior. J Neurosci.

[50] Schultz RT (2005). Developmental deficits in social perception in autism: the role of the amygdala and fusiform face area. Int J Dev Neurosci.

[51] Amaral DG (2002). The primate amygdala and the neurobiology of social behavior: implications for understanding social anxiety. Biol Psychiatry.

[52] Ferguson JN, Aldag JM, Insel TR, Young LJ (2001). Oxytocin in the medial amygdala is essential for social recognition in the mouse. J Neurosci.

[53] Phelps EA, LeDoux JE (2005). Contributions of the amygdala to emotion processing: from animal models to human behavior. Neuron.

[54] Paine TA, Swedlow N, Swetschinski L (2017). Decreasing GABA function within the medial prefrontal cortex or basolateral amygdala decreases sociability. Behav Brain Res.

[55] Meyer U (2013). Developmental neuroinflammation and schizophrenia. Prog Neuropsychopharmacol Biol Psychiatry.

[56] Meyer U, Feldon J, Dammann O (2011). Schizophrenia and autism: both shared and disorder-specific pathogenesis via perinatal inflammation?. Pediatr Res.

[57] Perry W, Minassian A, Lopez B, Maron L, Lincoln A (2007). Sensorimotor gating deficits in adults with autism. Biol Psychiatry.

[58] Oranje B, Lahuis B, van Engeland H, Jan van der Gaag R, Kemner C (2013). Sensory and sensorimotor gating in children with multiple complex developmental disorders (MCDD) and autism. Psychiatry Res.

[59] Kohl S (2014). Prepulse inhibition of the acoustic startle reflex in high functioning autism. Plos One.

[60] Takahashi, H. & Kamio, Y. Acoustic startle response and its modulation in schizophrenia and autism spectrum disorder in Asian subjects. *Schizophr Res*, 10.1016/j.schres.2017.05.034 (2017). 10.1016/j.schres.2017.05.034 28578923

[61] Sato W (2017). Reduced Gray Matter Volume in the Social Brain Network in Adults with Autism Spectrum Disorder. Frontiers in human neuroscience.

[62] Crawford JD (2015). Elevated GFAP Protein in Anterior Cingulate Cortical White Matter in Males With Autism Spectrum Disorder. Autism Res.

[63] Yui K, Sato A, Imataka G (2015). Mitochondrial Dysfunction and Its Relationship with mTOR Signaling and Oxidative Damage in Autism Spectrum Disorders. Mini reviews in medicinal chemistry.

[64] Gumusoglu SB, Fine RS, Murray SJ, Bittle JL, Stevens HE (2017). The role of IL-6 in neurodevelopment after prenatal stress. Brain, behavior, and immunity.

[65] Lee AS, Azmitia EC, Whitaker-Azmitia PM (2017). Developmental microglial priming in postmortem autism spectrum disorder temporal cortex. Brain, behavior, and immunity.

[66] Smith SE, Li J, Garbett K, Mirnics K, Patterson PH (2007). Maternal immune activation alters fetal brain development through interleukin-6. J Neurosci.

[67] Xin Y, Liu H, Zhang P, Chang L, Xie K (2017). Molecular hydrogen inhalation attenuates postoperative cognitive impairment in rats. Neuroreport.

[68] Zhang Y (2016). Effects of hydrogen-rich water on depressive-like behavior in mice. Sci Rep.

[69] Deng Y (2014). Astrocyte-derived proinflammatory cytokines induce hypomyelination in the periventricular white matter in the hypoxic neonatal brain. Plos One.

[70] Mallard C (2014). Astrocytes and microglia in acute cerebral injury underlying cerebral palsy associated with preterm birth. Pediatr Res.

[71] Penn AA, Gressens P, Fleiss B, Back SA, Gallo V (2016). Controversies in preterm brain injury. Neurobiol Dis.

[72] Chang E (2015). Preterm birth and the role of neuroprotection. BMJ (Clinical research ed.).

[73] Lai MC, Lombardo MV, Baron-Cohen S (2014). Autism. Lancet.

[74] Li H (2008). Transcription factor MEF2C influences neural stem/progenitor cell differentiation and maturation *in vivo*. Proceedings of the National Academy of Sciences of the United States of America.

[75] Ushida T (2016). Molecular hydrogen ameliorates several characteristics of preeclampsia in the Reduced Uterine Perfusion Pressure (RUPP) rat model. Free Radic Biol Med.

[76] Bhat MA (2001). Axon-glia interactions and the domain organization of myelinated axons requires neurexin IV/Caspr/Paranodin. Neuron.

[77] Chan YL (2016). Impact of maternal cigarette smoke exposure on brain inflammation and oxidative stress in male mice offspring. Sci Rep.

[78] Abbasi, Z., Behnam-Rassouli, F., Ghahramani Seno, M. M. & Fereidoni, M. A transient insulin system dysfunction in newborn rat brain followed by neonatal intracerebroventricular administration of streptozotocin could be accompanied by a labile cognitive impairment. *Neuroscience research*, 10.1016/j.neures.2017.10.003 (2017). 10.1016/j.neures.2017.10.003 29055675

[79] McMahon JJ (2015). Seizure-dependent mTOR activation in 5-HT neurons promotes autism-like behaviors in mice. Neurobiol Dis.

[80] Ibi D (2010). Combined effect of neonatal immune activation and mutant DISC1 on phenotypic changes in adulthood. Behav Brain Res.

[81] Hori K (2015). Heterozygous Disruption of Autism susceptibility candidate 2 Causes Impaired Emotional Control and Cognitive Memory. Plos One.

[82] Liang J (2008). Excitatory amino acid transporter expression by astrocytes is neuroprotective against microglial excitotoxicity. Brain Res.

[83] Suzumura A, Lavi E, Weiss SR, Silberberg DH (1986). Coronavirus infection induces H-2 antigen expression on oligodendrocytes and astrocytes. Science (New York, N.Y.).

