# Supplementary material for: Administration of molecular hydrogen during pregnancy improves behavioral abnormalities of offspring in a maternal immune activation model
Source: Sci Rep. 2018 Jun 15;8:9221. doi: 10.1038/s41598-018-27626-4 (PMC6003913; doi:10.1038/s41598-018-27626-4)
Supplement: Supplementary file 1 — Supplementary information [file 41598_2018_27626_MOESM1_ESM.pdf]

Supplementary information

Administration of molecular hydrogen during pregnancy improves behavioral abnormalities of offspring in a maternal immune activation model

Kenji Imai[a], Tomomi Kotani[a], Hiroyuki Tsuda[e], Tomoko Nakano[a], Takafumi Ushida[a], Akira Iwase[a], Taku Nagai[b], Shinya Toyokuni[c], Akio Suzumura[d], Fumitaka Kikkawa[a]

[a]Department of Obstetrics and Gynecology, [b]Department of Neuropsychopharmacology and Hospital Pharmacy, and [c]Department of Pathology and Biological Responses, Nagoya University Graduate School of Medicine, 65 Tsurumai-cho, Showa-ku, Nagoya 466-8550, Japan. [d]Department of Neuroimmunology, Research Institute of Environmental Medicine, Nagoya University, Furo-cho, Chikusa-ku, Nagoya 464-8601, Japan. [e]Department of Obstetrics and Gynecology, Japanese Red Cross Nagoya Daiichi Hospital, 3-35, Michishita-Cho, Nakamura-Ku, Nagoya, 453-8511, Japan.

Correspondence to: Tomomi Kotani, Department of Obstetrics and Gynecology, Nagoya University Graduate School of Medicine, 65 Tsurumai-cho, Showa-ku, Nagoya 466-8550, Japan, email: itoto@med.nagoya-u.ac.jp, Tel: +81-52-744-2261, Fax: +81-52-744-2268

Supplemental Method

DNA microarray

Changes in astrocytic gene expression associated with LPS or H_2 administration were examined. Primary astrocytes were isolated from newborn mice of each group; Control group, LPS group, and HW+LPS group, as described in the Materials and Methods. Total RNA from primary astrocytes of each group were prepared using the RNeasy Mini Kit. DNA microarray experiments were performed using an Agilent Expression Array Whole Mouse Genome oligo DNA microarray (Agilent, Santa Clara, CA) at Hokkaido System Science (Sapporo, Japan) with the microarray service certified by Agilent.

Fig. S1. Representative images of Nissl staining in the hippocampus. The number of neurons in the hippocampus was unchanged in the LPS-treated offspring compared with the Control group. LPS, lipopolysaccharide.

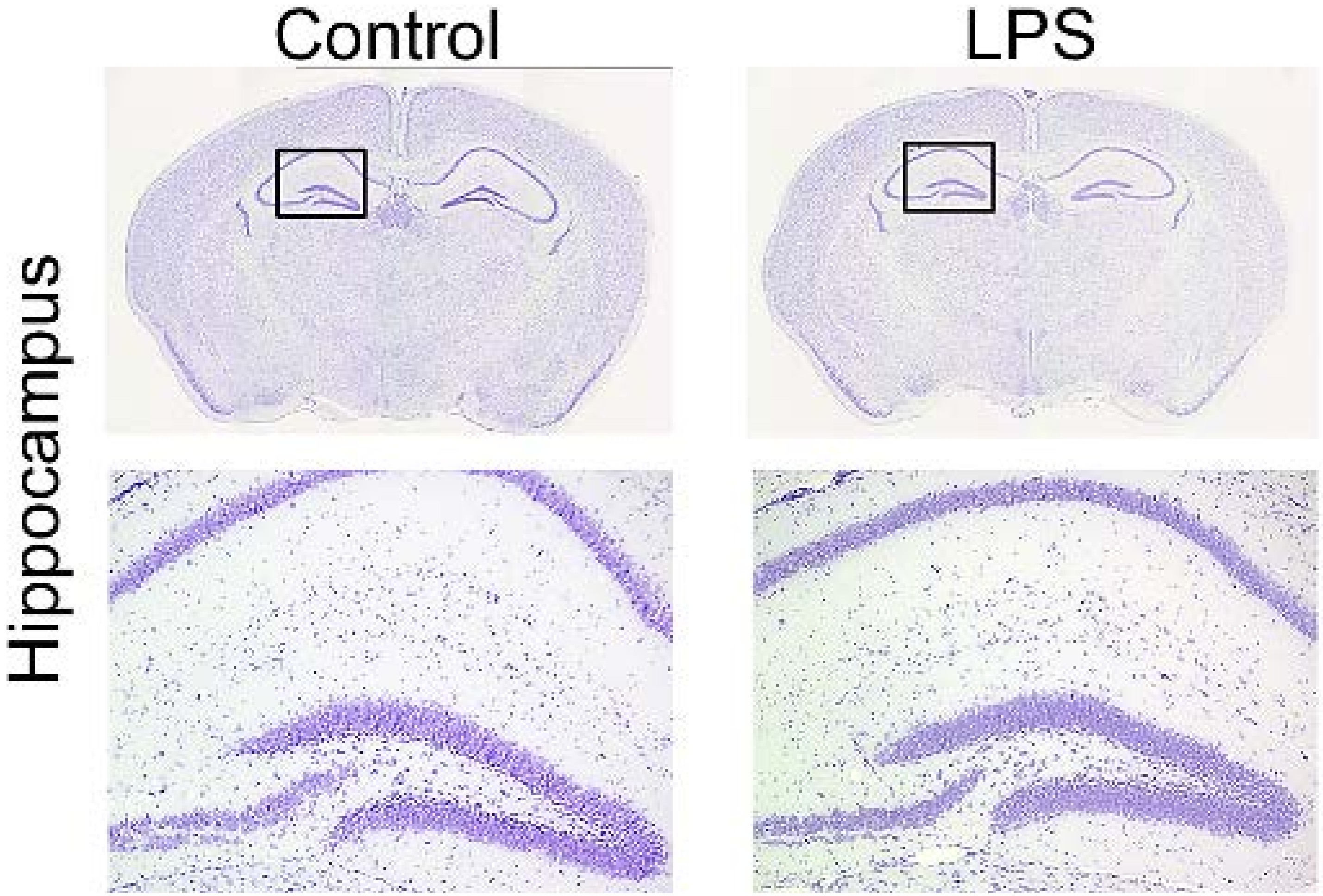

Table S1. Astrocytic genes up-regulated in LPS group compared with Control group

GeneName	Systematic Name	Description	FoldChange
Zfp608	AK047979	16 days embryo head cDNA, RIKEN full-length enriched library, clone:C130026F18 product:hypothetical Glycine-rich region containing protein, full insert sequence.	11.95
Prl2c5	NM_181852	prolactin family 2, subfamily c, member 5	11.71
Wnt10a	NM_009518	wingless-type MMTV integration site family, member 10A	8.36
2610305D13Rik	NM_145078	RIKEN cDNA 2610305D13 gene	8.33
Nell1	NM_001037906	NEL-like 1	7.43
Stc2	NM_011491	stanniocalcin 2	5.72
Crct1	NM_028798	cysteine-rich C-terminal 1	5.38
1700003E24Rik	NR_103799	RIKEN cDNA 1700003E24 gene, non-coding RNA	4.39
Xaf1	NM_001037713	XIAP associated factor 1, transcript variant 1	4.11
Myh8	NM_177369	myosin, heavy polypeptide 8, skeletal muscle, perinatal	4.11
Slit3	NM_011412	slit homolog 3 (Drosophila)	4.11
Il19	NM_001009940	interleukin 19	4.09
1700084M14Rik	NR_126536	RIKEN cDNA 1700084M14 gene, non-coding RNA	4.06
Tacr1	NM_009313	tachykinin receptor 1	4.04
1700019M22Rik	NR_103800	RIKEN cDNA 1700019M22 gene, non-coding RNA	4.03
Kcnn4	NM_008433	potassium intermediate/small conductance calcium-activated channel, subfamily N, member 4, transcript variant 1	4.03
BC024984	BC024984	cDNA clone IMAGE:5028619, partial cds.	3.98
Il7	NM_008371	interleukin 7	3.95
Anxa8	NM_013473	annexin A8, transcript variant 1	3.95
Aasdh	NM_173765	aminoadipate-semialdehyde dehydrogenase	3.94
Il19	NM_001009940	interleukin 19	3.94
Artn	NM_001284192	artemin, transcript variant 2	3.88
Il7r	NM_008372	interleukin 7 receptor	3.85
Mzt1	NM_175245	mitotic spindle organizing protein 1	3.78
Ccl20	NM_016960	chemokine (C-C motif) ligand 20, transcript variant 1	3.65
Kcnn4	NM_001163510	potassium intermediate/small conductance calcium-activated channel, subfamily N, member 4, transcript variant 2	3.63
Nkain2	NM_001013411	Na+/K+ transporting ATPase interacting 2, transcript variant 1	3.52
Adrb3	NM_013462	adrenergic receptor, beta 3	3.52
Pgam2	NM_018870	phosphoglycerate mutase 2	3.51
Cyp4f18	NM_024444	cytochrome P450, family 4, subfamily f, polypeptide 18	3.51
Foxf1	NM_010426	forkhead box F1	3.50
Gata3	NM_008091	GATA binding protein 3	3.49
Prex2	NM_029525	phosphatidylinositol-3,4,5-trisphosphate-dependent Rac exchange factor 2, transcript variant 1	3.49
Gm8909	NM_001081032	predicted gene 8909	3.37
Klhdc8a	NM_144810	kelch domain containing 8A	3.28
Itga3	NM_013565	integrin alpha 3, transcript variant 1	3.27
Cd34	NM_133654	CD34 antigen, transcript variant 2	3.25
Scn5a	NM_021544	sodium channel, voltage-gated, type V, alpha, transcript variant 1	3.18
Kcnip1	NM_001290690	Kv channel-interacting protein 1, transcript variant D	3.18
Sema3f	NM_011349	sema domain, immunoglobulin domain (Ig), short basic domain, secreted, (semaphorin) 3F	3.16
Wisp2	NM_016873	WNT1 inducible signaling pathway protein 2	3.08
Tspan11	NM_026743	tetraspanin 11	3.08
Rgs1	NM_015811	regulator of G-protein signaling 1	3.06
Scx	NM_198885	scleraxis	3.05
Prelp	NM_054077	proline arginine-rich end leucine-rich repeat	3.04
Cpxm2	NM_018867	carboxypeptidase X 2 (M14 family)	3.04
Prelp	NM_054077	proline arginine-rich end leucine-rich repeat	3.02
Slc25a17	NM_011399	solute carrier family 25 (mitochondrial carrier, peroxisomal membrane protein), member 17	3.01

Table S2. Astrocytic genes down-regulated in LPS group compared with Control group

GeneName	Systematic Name	Description	FoldChange
Qprt	NM_133686	quinolinate phosphoribosyltransferase	0.017
Serpina3g	NM_009251	serine (or cysteine) peptidase inhibitor, clade A, member 3G	0.025
Otor	NM_020595	otoraplin	0.026
Hp	NM_017370	haptoglobin (Hp)	0.036
Serpinb1c	NM_173051	serine (or cysteine) peptidase inhibitor, clade B, member 1c	0.040
Serpinb1a	NM_025429	serine (or cysteine) peptidase inhibitor, clade B, member 1a	0.041
Sfrp4	NM_016687	secreted frizzled-related protein 4	0.042
Cxcl12	NM_001012477	chemokine (C-X-C motif) ligand 12, transcript variant 3	0.045
Gjb2	NM_008125	gap junction protein, beta 2	0.047
Lgi2	NM_144945	leucine-rich repeat LGI family, member 2	0.055
Ccl8	NM_021443	chemokine (C-C motif) ligand 8	0.064
Gper1	NM_029771	G protein-coupled estrogen receptor 1	0.064
Efemp1	NM_146015	epidermal growth factor-containing fibulin-like extracellular matrix protein 1	0.074
Lrrc32	NM_001113379	leucine rich repeat containing 32	0.078
Dio2	NM_010050	deiodinase, iodothyronine, type II	0.079
Moxd1	NM_021509	monooxygenase, DBH-like 1	0.082
Efemp1	NM_146015	epidermal growth factor-containing fibulin-like extracellular matrix protein 1	0.086
Cxcl12	NM_021704	chemokine (C-X-C motif) ligand 12, transcript variant 1	0.092
Pear1	NM_028460	platelet endothelial aggregation receptor 1, transcript variant 1	0.096
Fbn2	NM_010181	fibrillin 2	0.098
Six2	NM_011380	sine oculis-related homeobox 2	0.098
5830417I10Rik	NR_028359	RIKEN cDNA 5830417I10 gene	0.098
Fbn2	NM_010181	fibrillin 2	0.098
9030619P08Rik	NR_108041	RIKEN cDNA 9030619P08 gene, non-coding RNA	0.100
Col1a2	NM_007743	collagen, type I, alpha 2	0.100
chr1:93812836-93816128_F	chr1:93812836-93816128_F	lincRNA:chr1:93812836-93816128 forward strand	0.105
Cpz	NM_153107	carboxypeptidase Z	0.110
Cited4	NM_019563	Cbp/p300-interacting transactivator, with Glu/Asp-rich carboxy-terminal domain, 4	0.111
Vtn	NM_011707	vitronectin	0.112
Tbx18	NM_023814	T-box18	0.113
Eng	NM_001146350	endoglin, transcript variant 2	0.113
Gm14625	NM_001220498	predicted gene 14625	0.115
Ptchd1	NM_001093750	patched domain containing 1	0.118
Gdf10	NM_145741	growth differentiation factor 10	0.119
Epha7	NM_001122889	Eph receptor A7, transcript variant 2	0.127
Ly6a	NM_010738	lymphocyte antigen 6 complex, locus A, transcript variant 3	0.132
Gas2	NM_008087	growth arrest specific 2	0.132
Rab15	NM_134050	RAB15, member RAS oncogene family	0.133
D630010B17Rik	NR_045629	RIKEN cDNA D630010B17 gene, long non-coding RNA	0.133
Ly6c2	NM_001099217	lymphocyte antigen 6 complex, locus C2	0.134
Adra2a	NM_007417	adrenergic receptor, alpha 2a	0.136
Ly6c1	NM_001252055	lymphocyte antigen 6 complex, locus C1, transcript variant 2	0.137
Ctsk	NM_007802	cathepsin K	0.137
Dcn	NM_007833	decorin, transcript variant 2	0.138
Mgp	NM_008597	matrix Gla protein	0.139
Adm	NM_009627	adrenomedullin	0.140

Table S3. Astrocytic genes up-regulated in HW + LPS group compared with LPS group

GeneName	Systematic Name	Description	FoldChange
Xaf1	NM_001037713	XIAP associated factor 1, transcript variant 1	5.19
Eif2s3y	NM_012011	eukaryotic translation initiation factor 2, subunit 3, structural gene Y-linked	4.99
Mmp8	NM_008611	matrix metallopeptidase 8	4.38
5830417I10Rik	NR_028359	RIKEN cDNA 5830417I10 gene, non-coding RNA	3.90
Tescl	NM_001163810	tescalcin-like	3.06
Slc6a12	NM_133661	solute carrier family 6 (neurotransmitter transporter, betaine/GABA), member 12	2.98
Ms4a4a	XM_006543453	ref|PREDICTED: Mus musculus membrane-spanning 4-domains, subfamily A, member 4A	2.84
Pf4	NM_019932	platelet factor 4	2.78
Stac2	NM_146028	SH3 and cysteine rich domain 2	2.60
Xlr4b	NM_021365	X-linked lymphocyte-regulated 4B, transcript variant 1	2.58
Plbd1	NM_025806	phospholipase B domain containing 1	2.54
Clec4n	NM_020001	C-type lectin domain family 4, member n, transcript variant 1	2.53
Saa2	NM_011314	serum amyloid A 2	2.53
Bst1	NM_009763	bone marrow stromal cell antigen 1	2.49
Pira2	NM_011089	paired-Ig-like receptor A2	2.39
Hist1h4d	NM_175654	histone cluster 1, H4d	2.38
AW551984	NM_001199556	expressed sequence AW551984, transcript variant 1	2.38
Ahrr	NM_009644	aryl-hydrocarbon receptor repressor	2.37
Entpd3	NM_178676	ectonucleoside triphosphate diphosphohydrolase 3	2.37
Lclat1	NM_001081071	lysocardiolipin acyltransferase, transcript variant 1	2.35
chr17:22420025-22457650_R	chr17:22420025-22457650_R	lincRNA:chr17:22420025-22457650 reverse strand	2.33
Fcmr	NM_026976	Fas apoptotic inhibitory molecule 3	2.28
H2-M2	NM_008204	histocompatibility 2, M region locus 2	2.27
Gm15698	NR_003564	predicted gene 15698, non-coding RNA	2.27
Coch	NM_007728	coagulation factor C homolog (Limulus polyphemus), transcript variant 1	2.26
Cxcl12	NM_021704	chemokine (C-X-C motif) ligand 12, transcript variant 1	2.26
Pcsk5	NM_001163144	proprotein convertase subtilisin/kexin type 5, transcript variant 2	2.24
Lyzl4	NM_026915	lysozyme-like 4	2.23
chr2:132079092-132087069_F	chr2:132079092-132087069_F	lincRNA:chr2:132079092-132087069 forward strand	2.23
Clec4b1	NM_027218	C-type lectin domain family 4, member b1, transcript variant 2	2.23
Mapk13	NM_011950	mitogen-activated protein kinase 13	2.23
Lcn2	NM_008491	lipocalin 2	2.22
Smoc2	AK040109	0 day neonate thymus cDNA, RIKEN full-length enriched library, clone:A430062I15 product:secreted modular calcium binding protein 2, full insert sequence	2.17
Pdgfd	ENSMUST00000058692	ens|platelet-derived growth factor, D polypeptide	2.17
Xlr3b	NM_001081643	X-linked lymphocyte-regulated 3B	2.16
Alox5	NM_009662	arachidonate 5-lipoxygenase	2.15
Hist1h4k	NM_178211	histone cluster 1, H4k	2.14
Pla2g2d	NM_011109	phospholipase A2, group IID	2.13
Pilrb1	NM_133209	paired immunoglobin-like type 2 receptor beta 1	2.11
Emb	NM_010330	embigin	2.06
Fxyd2	NM_052823	FXYD domain-containing ion transport regulator 2, transcript variant b,	2.05
Syt6	NM_018800	synaptotagmin VI, transcript variant 1	2.03
Tfec	NM_031198	transcription factor EC	2.03
Pira11	NM_011088	paired-Ig-like receptor A11	2.01
Gm14548	NM_001166672	predicted gene 14548	2.01

Table S4. Astrocytic genes down-regulated in HW + LPS group compared with LPS group

GeneName	Systematic Name	Description	FoldChange
Zranb2	NM_017381	zinc finger, RAN-binding domain containing 2	0.084
Lum	NM_008524	lumican	0.154
Igf2	NM_010514	insulin-like growth factor 2, transcript variant 1	0.222
Cxcl15	NM_011339	chemokine (C-X-C motif) ligand 15	0.224
Piezo2	NM_001039485	piezo-type mechanosensitive ion channel component 2	0.238
Odf3b	NM_001013022	outer dense fiber of sperm tails 3B	0.262
Plac9a	NM_207229	placenta specific 9a	0.267
Fam216b	NM_177629	family with sequence similarity 216, member B	0.282
Wnt6	NM_009526	wingless-type MMTV integration site family, member 6	0.291
Cdhr4	ENSMUST00000177226	ens|cadherin-related family member 4	0.304
St3gal3	NM_009176	ST3 beta-galactoside alpha-2,3-sialyltransferase 3	0.308
Cftr	NM_021050	cystic fibrosis transmembrane conductance regulator	0.314
Sdk1	NM_177879	sidekick homolog 1 (chicken)	0.314
Rarb	NM_001289760	retinoic acid receptor, beta, transcript variant beta3	0.314
Ucma	NM_026754	upper zone of growth plate and cartilage matrix associated, transcript variant 1	0.316
Bbox1	NM_130452	butyrobetaine (gamma), 2-oxoglutarate dioxygenase 1 (gamma-butyrobetaine hydroxylase)	0.318
Spon2	NM_133903	spondin 2, extracellular matrix protein	0.336
Mrgprf	NM_145379	MAS-related GPR, member F	0.337
Aldh1a2	NM_009022	aldehyde dehydrogenase family 1, subfamily A2	0.338
Olfml3	ENSMUST00000154145	ens|olfactomedin-like 3	0.339
Prss35	ENSMUST00000036426	ens|protease, serine 35	0.350
Ucma	NM_001113558	upper zone of growth plate and cartilage matrix associated, transcript variant 2	0.351
Xist	NR_001570	inactive X specific transcripts, transcript variant 2, long non-coding RNA	0.352
Epha3	NM_010140	Eph receptor A3	0.354
H19	NR_130973	H19, imprinted maternally expressed transcript, transcript variant 1, long non-coding RNA	0.361
Ntsr2	NM_008747	neurotensin receptor 2	0.362
Sema3d	NM_028882	sema domain, immunoglobulin domain (Ig), short basic domain, secreted, (semaphorin) 3D	0.366
XIST	XIST	lincRNA:chrX:100655711-100678572 reverse strand	0.370
Miat	NR_033657	myocardial infarction associated transcript (non-protein coding), transcript variant 1, long non-coding RNA	0.377
Col2a1	NM_031163	collagen, type II, alpha 1, transcript variant 1	0.378
Epha3	NM_010140	Eph receptor A3	0.382
Lect1	NM_010701	leukocyte cell derived chemotaxin 1	0.382
Mchr1	NM_145132	melanin-concentrating hormone receptor 1	0.383
Pifo	NM_029604	primary cilia formation, transcript variant 2	0.385
Npy	NM_023456	neuropeptide Y	0.388
Mid1	NM_001290505	midline 1, transcript variant 4	0.391
Vill	NM_001164567	villin-like, transcript variant 1	0.392
Rarb	NM_011243	retinoic acid receptor, beta, transcript variant beta2	0.395
Fhad1	NM_177868	forkhead-associated (FHA) phosphopeptide binding domain 1	0.396
Ablim3	NM_198649	actin binding LIM protein family, member 3, transcript variant 1	0.398
B430306N03Rik	NM_177083	RIKEN cDNA B430306N03 gene	0.402
Fbln1	NM_010180	fibulin 1	0.405
Tekt1	NM_001282006	tektin 1, transcript variant 1	0.406
H2-Eb1	NM_010382	histocompatibility 2, class II antigen E beta	0.408
Edil3	NM_010103	EGF-like repeats and discoidin I-like domains 3, transcript variant 2	0.410
Cmtm5	NM_026066	CKLF-like MARVEL transmembrane domain containing 5, transcript variant 2	0.410
Fgf7	NM_008008	fibroblast growth factor 7	0.411
